# Machine Learning-Driven Data Fusion of Chromatograms, Plasmagrams, and IR Spectra of Chemical Compounds of Forensic Interest

**DOI:** 10.1021/acsomega.4c10107

**Published:** 2025-02-11

**Authors:** Giorgio Felizzato, Giuliano Iacobellis, Nicola Liberatore, Sandro Mengali, Martin Sabo, Patrizia Scandurra, Roberto Viola, Francesco Saverio Romolo

**Affiliations:** † University of Bergamo, Via Moroni 255, Bergamo 24127, Italy; ‡ Raggruppamento Carabinieri Investigazioni Scientifiche, Reparto Ricerca e Sviluppo of Rome, Viale di Tor di Quinto, 119, Rome 00191, Italy; § Consorzio CREO, L’Aquila 67100, Italy; ∥ MaSa Tech, s.r.o., Sadová 3018/10, Stará Turá 916 01, Slovakia; ⊥ Faculty of Informatics and Information Technologies, Slovak University of Technology in Bratislava, Ilkovičova 2, Bratislava 4 842 16, Slovakia

## Abstract

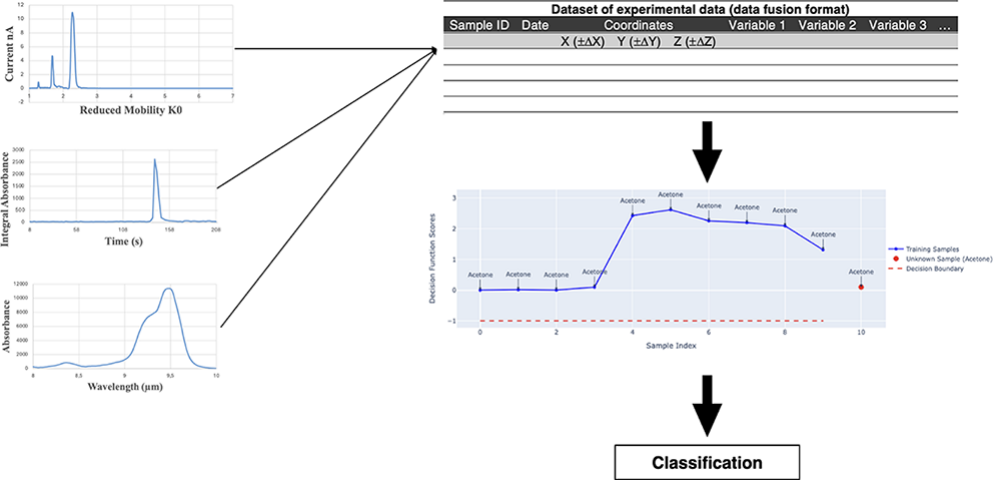

Achieving fast analytical results on-site with the highest possible accuracy in forensic analyses is crucial for investigations. While portable sensors are essential for crime scene analysis, they often face limitations in sensitivity and specificity, especially due to environmental factors. Data fusion (DF) techniques can enhance accuracy and reliability by combining information from multiple sensors. This study develops different DF approaches using data from two sensors: ion mobility spectrometry (IMS) and gas chromatography-quartz-enhanced photoacoustic spectroscopy (GC-QEPAS), aiming to improve the safety of crime scene operators and the accuracy of on-site forensic analysis. Two DF approaches were developed for acetone and DMMP: low-level (LLDF) and mid-level (MLDF), meanwhile a high-level (HLDF) approach was applied to TATP. LLDF concatenated preprocessed data matrices, while MLDF employed principal component analysis for feature extraction. LLDF and MLDF used one-class support vector machines (OC-SVM) for classification, while HLDF combined OC-SVM for IMS and SIMCA for GC-QEPAS. Sensor location within crime scenes was established using traditional measuring tape and laser distance meters, with a 1 m cutoff distance between sensors deemed appropriate for indoor crime scenes. LLDF achieved high accuracy but was sensitive to concentration variations, while MLDF enhanced the classification robustness. HLDF allowed for independent sensor use in real scenarios. All of the methods reached 100% accuracy for DMMP and acetone, and the MLDF approach was the fastest among the DF methods, demonstrating its potential for rapid applications. DF approaches can significantly enhance the safety and accuracy of forensic investigations, with future research planned to extend data sets and include more sensors.

## 1 Introduction

Crime scene investigation (CSI) involves various specialists’ coordinated efforts, including first responders, health personnel, police forces, and forensic scientists. When arriving at the crime scene, the safety and security of people (both victims and operators) is the priority, and rescue of injured or intoxicated victims must be guaranteed in a timely manner. As a result, it is crucial to promptly assess the presence of hazardous materials.^1−3^ This is done using analytical tools, such as field kits and portable detectors.

Different portable detectors have specific features and are expected to provide diverse chemical information on the same sample. Appropriately mixing the analytical signals could lead to a synergistic answer, improving the accuracy of exploratory and predictive models, such as classification or quantitative ones. This integrated strategy is known as data fusion (DF).^4−6^

DF can be performed only when different instruments analyze the same sample or specimen. However, in crime scene investigations, this task is particularly challenging because sensors are typically moved around in search of traces to be analyzed. Traditionally, the positions of traces within the crime scene have been determined using a measuring tape, which aids in the creation of crime scene sketches.^7^ According to the ENFSI Best Practice Manual for Crime Scene Investigation^8^ and the ENFSI Best Practice Manual for the Implementation of a Quality Management System and Accreditation Model for Crime Scene Investigation,^9^ before analyzing any traces at a crime scene, the exact locations of sensors must be recorded using either a measuring tape or electronic measuring devices. In the context of sensor DF in forensic applications, instruments must analyze the same trace at a specific location within the crime scene. Consequently, the position of the instrument during sampling must be precisely recorded to ensure accuracy of the DF process. Moreover, if sensors analyze the same area at different times, then the reliability of the data fusion process may be compromised, as the cloud of chemical substances can disperse over time.

In this article, a novel approach to machine learning-driven data fusion of analytical results from portable sensors for forensic applications is described, particularly focusing on operators’ safety, by considering optimal positioning of sensors within the crime scene. Based on the already published works in the literature, we developed three different expert systems: based on low-level DF (LLDF), mid-level DF (MLDF), and high-level DF (HLDF), to enhance the identification accuracy for crime scene investigation.

For this purpose, several DF modules have been developed by employing two analytical sensors from the RISEN project (HORIZON2020, Grant Agreement No. 883116): an IMS sensor by MaSaTECH (Slovakia)^10^ and a GC-QEPAS by Consorzio CREO (L’Aquila, Italy).^11,12^ In the early stage of the study, the experimental data from the two sensors were studied and preprocessed to enhance reliability. Furthermore, various classification methodologies for each sensor were investigated to offer a comprehensive comparison between DF approaches and the performance of the sensors used alone. Following this step, the DF approach was focused on automating the classification task based on the sensors’ locations (their relative coordinates) within the crime scene. In this study, various machine learning (ML) methods were employed as tools for automating classification, including the one-class support vector machine (OC-SVM), the soft independent modeling of class analogy (SIMCA), and a combination of feature extraction using principal component analysis (PCA) with OC-SVM. In addition, the CPU time for each machine learning model was assessed to ensure the efficiency of our approaches in forensic scenarios. Indeed, in forensic investigations, the ability to quickly analyze data is just as important as accuracy, ensuring that timely decisions can be made based on the findings, especially in safety applications.

The focus of the study was not the use of a large set of target analytes but rather the development of a new DF approach for real applications. The study has proven to be effective, opening new possibilities for improving safety and security during crime scene investigations.

## 2 Related Work

Expert systems based on ML methodologies have gained significant interest in recent years for a variety of applications. However, there is a lack of studies focusing on crime scene investigations and, more broadly, on-field applications.

Wenjun^13^ developed a sensor DF approach to characterize multiple soil properties by employing various analytical sensors. Since no single sensor can capture all relevant soil attributes, the study introduced a methodology for sensor DF and tested different sensor combinations to develop predictive models. The results demonstrated that sensor DF performed better than individual sensors in predicting soil properties, highlighting its potential to improve prediction accuracy. Additionally, geospatially correlated data was employed to better represent the spatial complexity of soils, further enhancing its characterization.

Ferrari et al.^6^ investigated a wireless sensor network designed to detect precursors of improvised explosives (IEs), such as hydrogen peroxide,^14^ in the area of explosives manufacturing sites. This network enabled early threat detection by employing an expert system, which could identify an explosive precursor either based on the signal from a single sensor or through data fusion from multiple sensors. By merging the responses from individual sensors, the system provided the end-user with an overall alarm level that represented a potential criminal threat of IE production. In this context, sensor location was a key factor; only outputs from sensors within user-defined proximity were integrated, ensuring timely and reliable threat detection.

Our study aims to address a gap in the state of the art, as no comprehensive study on expert systems for crime scene investigation has been published to date. While expert systems have been proven effective in various domains, their application in forensic science, particularly in crime scene investigations, remains almost unexplored, even though it has the potential to enhance the analysis and interpretation of evidence. While other studies have acknowledged the spatial positioning of sensors, none have specifically considered their relative positions within a defined area, such as a crime scene. Given that forensic investigations must follow strict guidelines, rules, and legal standards, this study emphasizes the importance of sensor placement to ensure the integrity of the evidence-collection process. This data fusion approach enhances the current state of the art by being applicable in real cases.

Moreover, based on laboratory experiments and data analysis, we have conducted a comprehensive evaluation of the sensors’ accuracy and the expert systems to provide a critical quantitative assessment of the proposed approach.

## 3 Background Concepts

**Figure 1:**
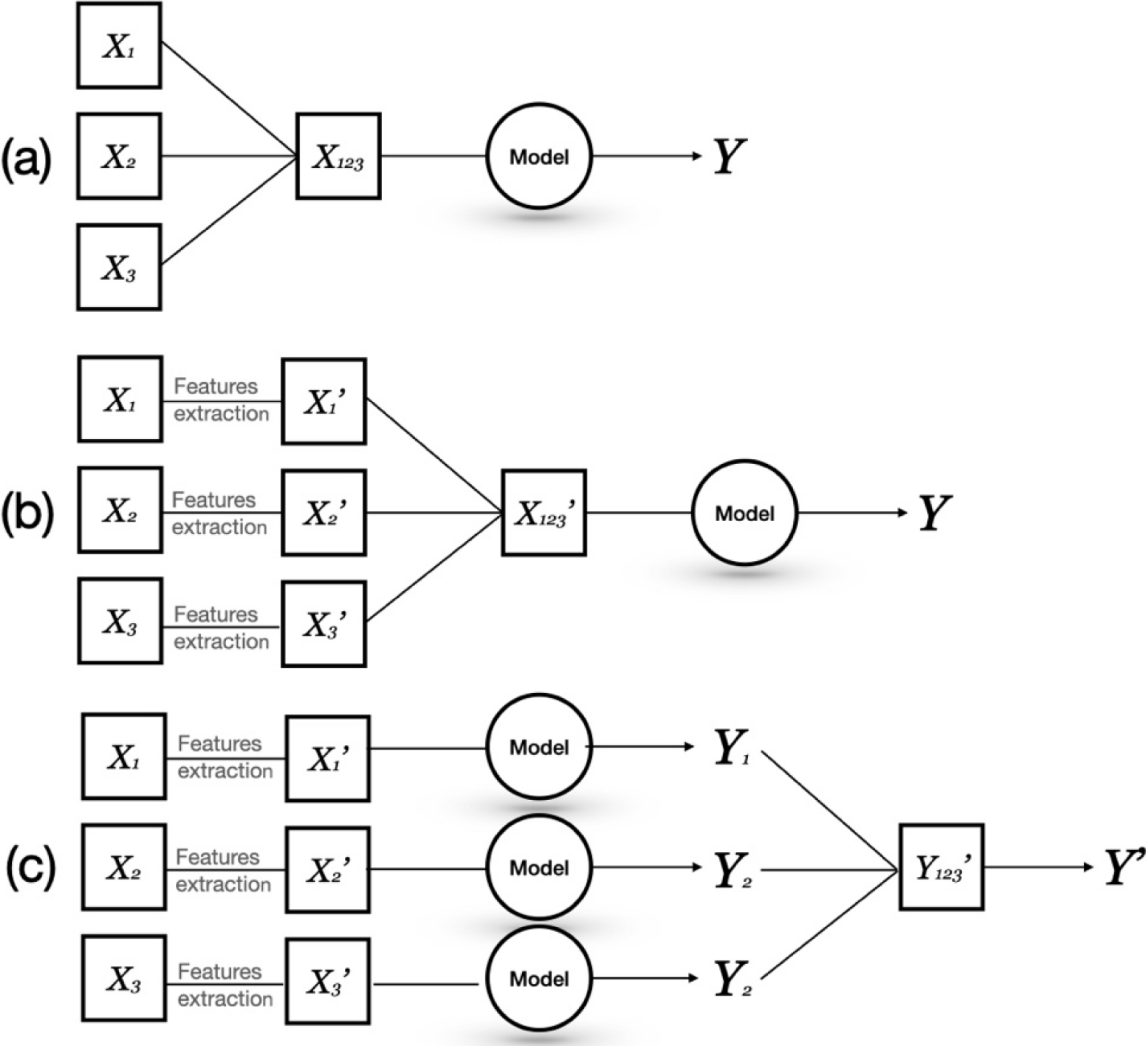
DF strategies: (a) LLDF, (b) MLDF, (c) HLDF.

DF strategies can be divided into three different classes based on the level at which the data are fused (Figure 1). In the LLDF, data from different sources are rearranged into a new data matrix, which will be the sum of the previously separated data sets. In MLDF, features are extracted from each data matrix, reducing its dimensionality and removing the noninformative variables. Then, features from different data matrices are merged into one single matrix. Finally, the HLDF works at the decision level, combining the classification results of each ML model involved.^15,16^

For the LLDF and MLDF, the OC-SVM model has been employed in this study because it has been proven effective in handling high-dimensional data sets and is flexible in defining the boundary of normal data points.^17,18^ One-class classification aims to identify a specific category of objects (inliers) while treating all others as outliers.^19^ The OC-SVM classifier achieves this by using a decision function that calculates the signed distance of each compound from the separating hyperplane: a positive distance indicates an inlier, while a negative distance indicates an outlier. When a compound is classified as an inlier, its characteristics align with those of the target compounds used to train the model, enabling its detection or identification. Furthermore, for the HLDF approach, the OC-SVM has been chosen for the IMS sensor, whereas the SIMCA model has been employed for the GC-QEPAS. The latter method is very useful for classifying high-dimensional observations because it incorporates PCA for dimension reduction. The basis of SIMCA is applying PCA to each group and then retaining a sufficient number of principal components in each group to calculate the largest variance. The result of SIMCA is a classification table in which an observation can be classified in one, or several classes, or not classified into any class.^20^

## 4 Materials and Methods

### 4.1 Target Compounds

In this study, three chemical compounds have been chosen to develop the DF modules and test their effectiveness: the sarin simulant dimethyl methylphosphonate (DMMP, CAS 756-79-6, Monforte Lab Suppliers, Grassobbio, Italy),^21^ acetone (CAS 67-64-1, Monforte Lab Suppliers, Grassobbio, Italy), a category 3 drug precursor based on EU regulation,^22^ and triacetone triperoxide (TATP, CAS 1336-17-0, supplied by Reparto Carabinieri Investigazioni Scientifiche of Rome), an improvised explosive used in many recent terrorist attacks.

### 4.2 Instruments

The IMS is integrated into a protective case together with a long-lifetime battery (6 h of work) and a small membrane pump (Pfeiffer Vacuum, Austria). The pump maintains the IMS drift tube at a subatmospheric pressure of 600 mbar, enabling continuous aspiration of environmental air. A laser diode (LD) module with a wavelength of 532 nm (green) and a power of 1 W is placed in front of the sniffing capillary. The focused laser beam promotes sample evaporation, which is immediately aspirated and analyzed by the IMS. The sensor operated in negative polarity, and it was used to analyze volatile compounds present in the environment.

The GC-QEPAS is made of three main components: (1) a gas sampler and preconcentrator designed for large air volumes: a compact purge and trap device utilizing commercial sorbent tubes from Markes International Ltd. This device can sample approximately 1 L of air and transfer preconcentrated vapors to the fast GC separation module in under 3 min. (2) A fast GC (CNR-IMM, Bologna, Italy)^23^ consisting of a micro-electro-mechanical system (MEMS) for preconcentration and injection, and an MEMS GC column for separation, both integrated on silicon micromachined chips. (3) A QEPAS detector measures the photoacoustic spectra of analytes eluted by the fast GC. This detector incorporates a quantum cascade laser source (MiniQCL, Block Engineering, USA) that continuously scans the thermal IR spectrum, covering wavelengths from 7.4 to 10.7 μm for spectroscopic analysis.

### 4.3 Data Fusion Implementation Details

The proposed multivariate data analysis was performed using a Python module within a Jupyter Notebook App, running on an Apple Mac Mini equipped with an eight-core CPU, a 10-core GPU, and 8 GB of unified memory. The following Python libraries for data analytics were used: NumPy,^24^ Pandas,^25^ Matplotlib,^26^ Plotly,^27^ and scikit-learn.^28^ The data from sensors were collected in a private repository on GitHub,^29^ where the DF modules were also deployed. This approach enabled fast work with the goal of automating the process in the future.

### 4.4 Sensor Positioning Method

The location of the sensors within the crime scene was measured using a measuring tape. Using a reference point in the crime scene, designated as point 000, the position of the sensor along the three Cartesian axes *x*, *y*, and *z* was determined. For each axis, the associated error was defined, which is essential for the second part of the DF. In this specific case, the experimental error was assumed to be minimum measurable distance with a measuring tape, which is 0.01 m. Furthermore, the sensor locations were measured by employing laser distance meters (Bosch GLM40). A laser distance meter can be positioned at the crime scene at coordinate 000 and rotated on itself to point the laser beam at the target, in this case, the sensors. As a result, the measurements should be less affected by human error.^30^ For the laser distance meter, the assumed experimental error is 0.0015 m according to the user manual. Measurements were taken using both a measuring tape and a laser distance meter from the reference point 000 to the sampler of each sensor.

Only when the distance is below a specific cutoff, the data fusion module concatenates the desired data matrices into a single one to provide the classification by means of the machine learning model. The distance among the sensors on the three axes (*x*, *y*, and *z*) has been calculated through the following formula:
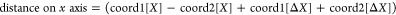
1
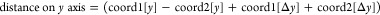
2
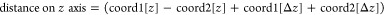
3
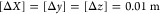
4

**Figure 2:**
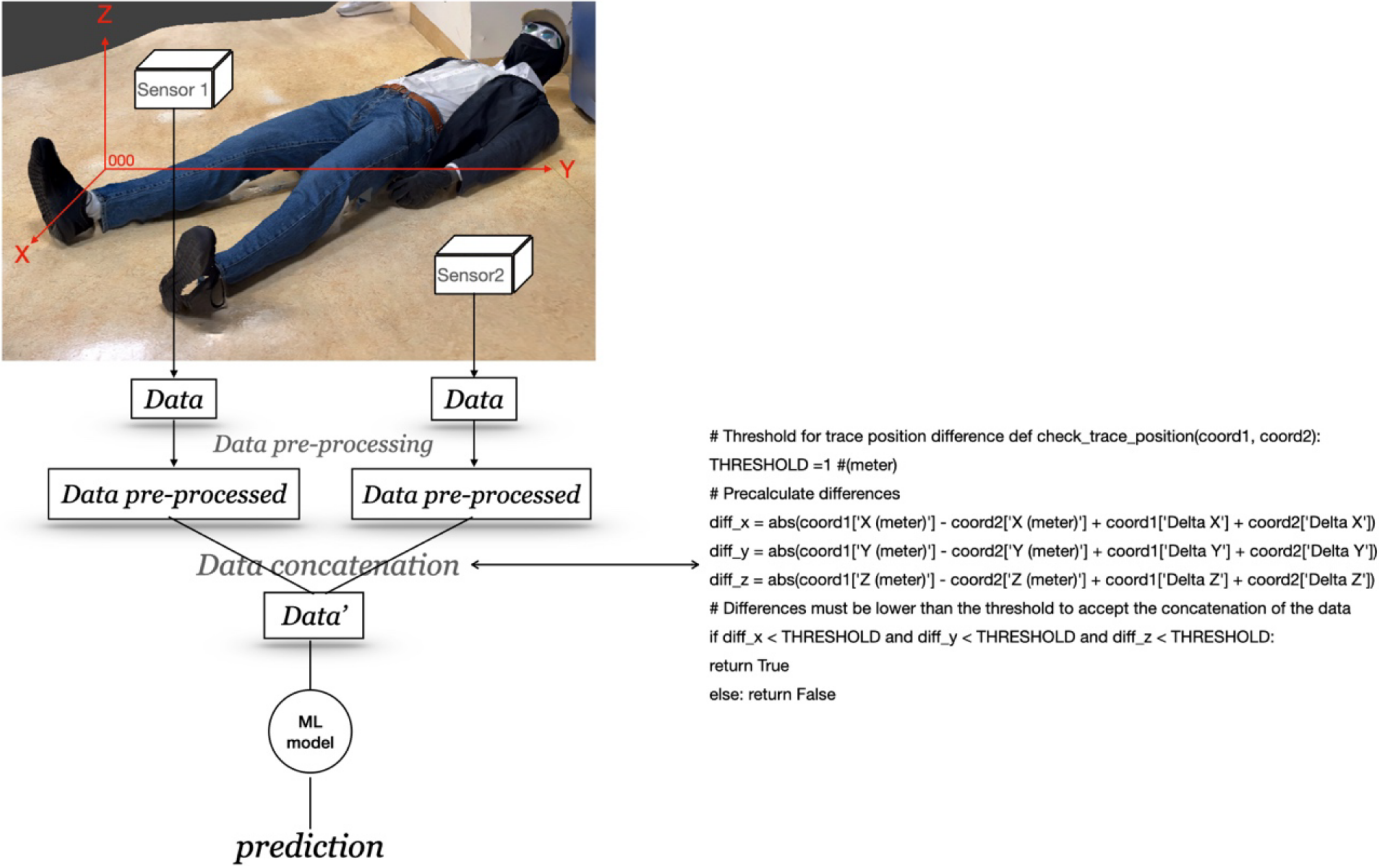
Mock crime scene along with sensor positions and reference point 000 for LLDF and MLDF.

Where coordinate 1 represents the coordinates of the first sensor, and coordinate 2 represents the coordinates of the second sensor (graphical example is shown in Figure 2). For safety applications, the threshold has been defined as 1 m long in the three coordinates. For an outdoor scenario, the threshold should be decided also considering the environmental conditions, such as wind speed and its direction. In the present study, only the indoor scenario has been considered.

### 4.5 Data Fusion of Acetone and DMMP Methodology

The LLDF and MLDF have been employed for acetone and DMMP using the IMS and GC-QEPAS sensor data.

**Figure 3:**
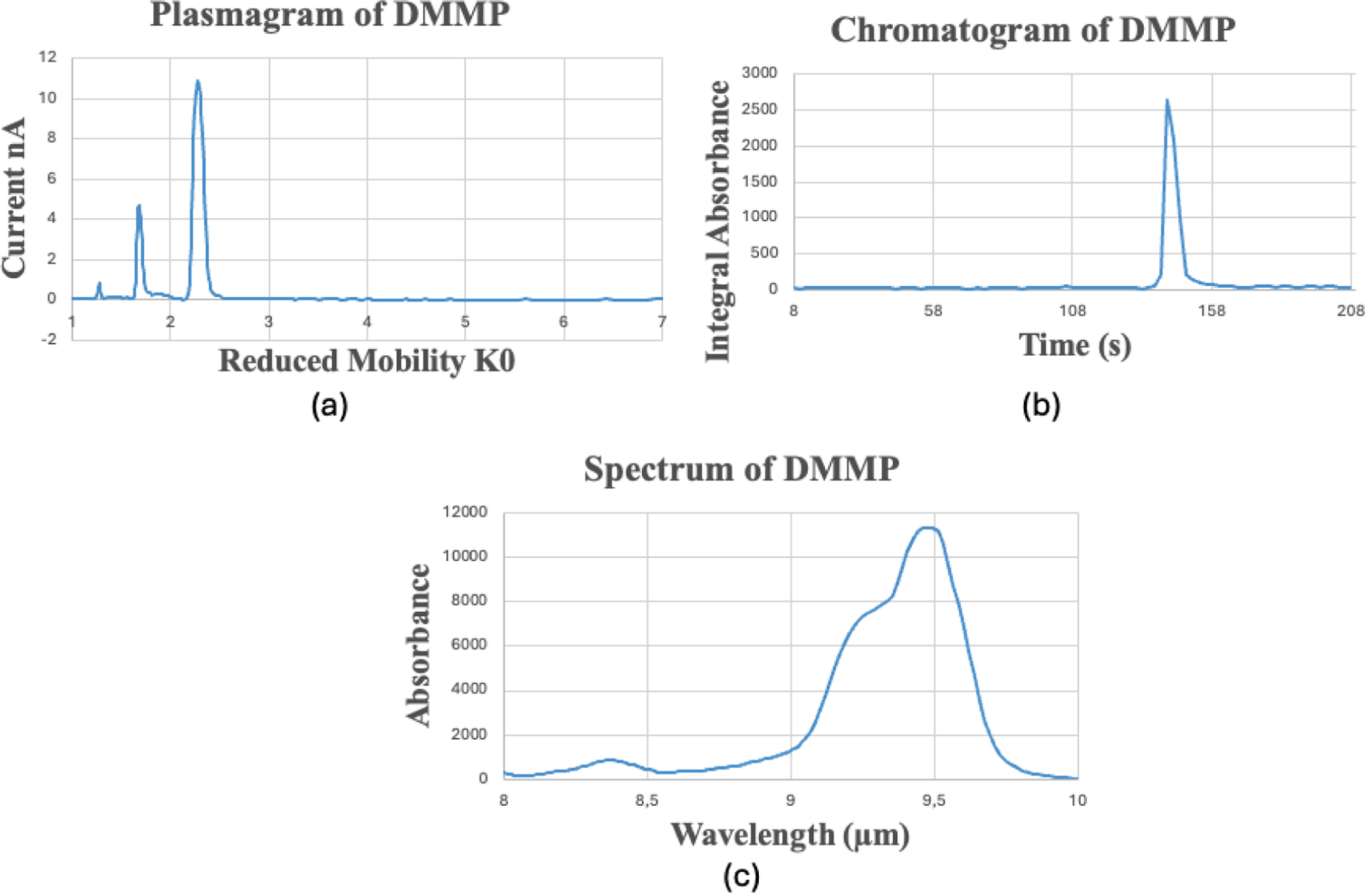
(a) Plasmagram of DMMP, (b) chromatogram of DMMP, and (c) IR spectrum of DMMP.

The IMS sensor employed in this article provided plasmagrams, as shown in Figure 3a. The GC-QEPAS sensor recorded chromatograms (e.g., see Figure 3b) and IR spectra (e.g., see Figure 3c).

The LLDF of DMMP and acetone was carried out by using the OC-SVM. To build a robust classification model, various analytical conditions were considered and varied. Indeed, four sample concentrations were used to vary the output signals of the sensors. Additionally, different operators analyzed the samples on different days and used different batches of solvents. As a result, the outputs considered are expected to vary, simulating real-world conditions for application of the method.

The first step of the DF was preprocessing of the experimental data to address variations in feature properties within a data set.^31^ The IMS data underwent autoscaling preprocessing, and the IR spectra underwent standard normal variate preprocessing. The retention times (RT) were used without any preprocessing method.

The preprocessed data for the LLDF have been concatenated to establish a single data matrix to train the OC-SVM model. Polynomial, sigmoid, linear, and radial basis kernel functions of the OC-SVM model were evaluated during the DF development. Based on the training data set, the boundary of the decision function was evaluated to avoid false negatives, which is particularly dangerous in safety applications for operators.

For the MLDF, the preprocessed data from the IMS and GC-QEPAS sensors underwent a feature extraction process by PCA. To reduce the dimensionality of the existing data matrices without losing important information, 99% of the explained variance was chosen as the criterion for selecting the number of principal components. The principal components of both data sets (IMS data and GC-QEPAS data) were concatenated into a single data matrix along with RTs. The resulting data matrix has been used to train the OC-SVM, based on four diverse kernel functions: polynomial, sigmoid, linear, and radial basis. Even for the MLDF, a specific boundary for the decision function was settled in order to avoid false negatives.

The parameters for the estimator, for both the LLDF and the MLDF, were obtained with the “model.get_params” function, which is part of the scikit-learn library, specifically for the SVM algorithm. This function is used to retrieve the hyperparameters of the SVM model, allowing users to inspect or modify the parameters that control the behavior of the model.

More details about the parameter settings are shown in Table 1.

**Table 1:** Parameters of OC-SVM for Both the DF Approaches

Parameters	Value
Cache_size	200
Coef0	0
Degree	3
Gamma	Auto
Kernel	Linear
Max_iter	–1
Tol	0.001
Verbose	False

### 4.6 Data Fusion of TATP Methodology

An HLDF approach was chosen for the DF of the TATP data. The identification of TATP by IMS was computed in two different ways. The first one is based on reduced mobility (RM) of the molecule, 2.06 cm^2^ V^–1^ s^–1^ and considers an uncertainty within 2%.^32^ A positive identification of TATP was confirmed when a peak with an intensity greater than 1 nA was detected within the specified reduced mobility range. Moreover, the OC-SVM model was performed using the TATP data recorded by IMS. A boundary threshold was established at −0.5, and a positive identification was achieved when the decision function value for the newly modeled data exceeded this threshold.

Finally, the GC-QEPAS data were used to train the SIMCA model. A positive identification with this ML approach was achieved when the newly modeled sample had a lower Euclidean distance and Mahalanobis distance to the centroid of the TATP class, defined during the training phase. In this study, only the safety application of the method has been taken into account: the Euclidean distance has been defined as equal to 5.5, meanwhile, the Mahalanobis distance has been fixed as 6.5 for positive identification.

## 5 Experimental Evaluation

This section reports on the evaluation of the proposed DF approach. To allow replication and verification of our results, a package (source code and data set) is publicly available in our GitHub repository.

### 5.1 Evaluation Design and Metrics

To validate the proposed method, we conducted experiments on each sensor involved in this article and on the ML system that was developed. For this purpose, we adopted metrics commonly used for evaluating the performance of classification methods: precision, recall, *F*1-score, and the overall model’s accuracy.^33^ Additionally, we evaluated the CPU times taken by the classifiers for training and analysis.

The workflow for the evaluation first considered each sensor individually, comparing different methodologies for each one. The evaluation of the IMS sensor has been conducted by employing two distinct approaches: identification through reduced mobility (RM) and the LDA model. Table 2 summarizes the number of experimental data used to evaluate the IMS system.

**Table 2:** Summary of Experimental Compounds for IMS Analysis

Compound	Number of experimental data
Acetone	16
DMMP	14
TATP	14

For the GC-QEPAS sensor, the evaluation of the system was conducted separately for the RT and IR spectra. To analyze RT, the probability density function (PDF) was employed to detect the target compounds. A positive detection was reached for a compound when the RT dwelled within
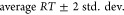
5

For the analysis of IR spectra, two distinct approaches were employed: Pearson’s correlation coefficient, in which a high score near 1 indicates high similarity, while a score near zero indicates that the two objects are dissimilar, and the LDA machine learning model.^34,35^

In addition, for the GC-QEPAS sensor, a DF process was performed by integrating the RT with IR spectra. First, the PDF and Pearson’s correlation were applied sequentially; second, an LLDF was employed by merging the RT and the corresponding IR spectra. The resulting data matrix was used to perform the LDA algorithms. Table 3 summarizes the compounds used to evaluate the different classification models. Acetone, DMMP, and TATP were further investigated as part of the DF process (Sections 4.5 and 4.6).

**Table 3:** Summary of Experimental Compounds for GC-QEPAS Analysis

Compound	Number of experimental data
Acetone	49
Benzaldehyde	13
BMK	9
DMMP	58
DPGME	41
Methyl salicylate	19
Safrole	13
Acetic acid	14
Toluene	19
TATP	16
DEMP	32
TMP	25
DES	30
Piperidine	29
MEK	28
Butyric acid	28
Tert-butyl methyl ether	23

Finally, data from both the IMS and GC-QEPAS sensors were merged to perform a comprehensive DF, as outlined in Sections 4.5 and 4.6. The ML models have been assessed using the data of acetone, DMMP, and TATP from both GC-QEPAS and IMS.

### 5.2 Results and Discussion

The IMS achieved high accuracy using both the RM and LDA as classification models. For the RM model, two distinct approaches were evaluated. The first approach considered the main peak of each analyte, while the second included two analytical peaks for DMMP. Table 4 shows both methodologies’ precision, recall, and *F*1-score for the three target compounds of the DF. When two peaks were considered for DMMP, its recall and *F*1-score dropped significantly. This is because the second DMMP peak appears only at high compound concentrations, leading to an increase in false negatives at lower concentrations. However, precision increased as the method became more selective, reducing false positives. The overall model accuracy for both methods was high, with values of 0.91 when a single peak was used for DMMP and 0.86 when using two peaks.

**Table 4:** Performance Parameters for the Two Applications of Reduced Mobility and LDA as a Classification Model for IMS Sensor

	Classification based on RM						LDA		
	Precision	Recall	*F*1-scores	Precision	Recall	*F*1-scores	Precision	Recall	*F*1-scores
	Considering 1 peak for DMMP			Considering 1 peak for DMMP					
Acetone	1	0.81	0.90	1	0.81	0.90	1	0.93	0.96
DMMP	0.78	1	0.88	1	0.43	0.60	1	1	1
TATP	1	0.64	0.78	1	0.64	0.78	0.94	1	0.97
Model accuracy			0.91			0.86			0.98

When the LDA model was used, accuracy increased significantly, reaching 0.98. Based on these results, LDA appears to be less affected by variations among the plasmagrams and deals with noise and low concentrations better than RM. Table 4 shows the performance parameters of this approach.

For the GC-QEPAS, initially, its two components were considered separately. The GC was employed to detect substances based on eq 5. However, the accuracy achieved with this method was 0.63, indicating that GC alone is insufficient for classification purposes.

For the QEPAS spectra, two classification models were applied: Pearson’s correlation method, which compares the experimental spectra with a reference spectrum, and the LDA model. Table 5 shows the performance metrics for both methods.

**Table 5:** Performance Parameters for the Pearson’s Correlation and LDA Model Based on QEPAS Spectra

	Pearson’s correlation			LDA		
	Precision	Recall	*F*1-score	Precision	Recall	*F*1-score
**Acetone**	0.82	0.92	0.87	0.96	1	0.98
**DMMP**	0.53	0.98	0.69	0.91	1	0.95
**TATP**	0.82	0.88	0.85	1	1	1
**Model accuracy**			0.82			0.90

The accuracy of the LDA method, calculated across all the analytes in Table 3, reached 0.90, compared to 0.82 for Pearson’s correlation. Furthermore, precision, recall, and *F*1-score were higher for the LDA model than for Pearson’s correlation across all target compounds.

Finally, RT and IR spectra were combined to improve the overall accuracy. Initially, the PDF method was applied prior to Pearson’s correlation approach, resulting in an accuracy increase to 0.99. This method significantly reduced both false positives and false negatives. Additionally, RT and IR spectra were integrated using a low-level data fusion approach with the LDA model, achieving an accuracy of 0.97. Table 6 summarizes the performance metrics for the DF of RT and IR spectra.

**Table 6:** Performance Parameters for the DF among Retention Times and QEPAS Spectra and among GC-QEPAS and IMS Sensors

	DF of RT and QEPAS spetctra						DF among IMS and GC-QEPAS		
	Precision	Recall	*F*1-score	Precision	Recall	*F*1-score	Precision	Recall	*F*1-score
	Pearson’s correlation			LDA					
Acetone	0.92	0.92	0.92	0.98	0.98	0.98	1	1	1
DMMP	0.93	0.91	0.92	0.93	0.97	0.95	1	0.93	0.96
TATP	1	0.88	0.93	1	1	1	1	1	1
Model accuracy			0.99			0.97			1

Despite achieving high accuracy, the LDA was less effective in modeling the RT and IR spectra using the LLDF approach compared to analyzing the two data sets separately through the PDF and Pearson’s correlation methods.

For the LLDF and the MLDF of acetone and DMMP, based on the model’s accuracy, the linear kernel proved to be the most effective in recognizing the inliers. To avoid false negatives, a threshold of the decision function values was established for the LLDF approach. In this case study of DMMP, a threshold of 0.0 was set. The parameter that most significantly affected the decision function value was the concentration of the samples based on the decision function values calculated for the samples of the training data set. Indeed, samples with the lowest concentrations were closer to the decision limit, whereas those with higher concentrations were farther from it. By means of cross-validation, employing the *k*-fold method, no false negatives were obtained using the threshold of 0.0, even for the samples with the lowest concentration (2.8 and 5.7 ppb), reaching an overall model’s accuracy of 100%. Table 6 summarizes the performance parameters for all of the target compounds in the DF.

Furthermore, a threshold of 0.0 was set for the decision function values of acetone. As observed earlier, samples with the lowest concentrations tended to dwell near the decision boundary. Setting this threshold allows one to limit false negatives as much as possible, ensuring that all positive samples are correctly classified. Employing the *k*-fold cross-validation method, using the threshold of 0.0, no false negatives were obtained, reaching again an overall model’s accuracy of 100%.

**Figure 4:**
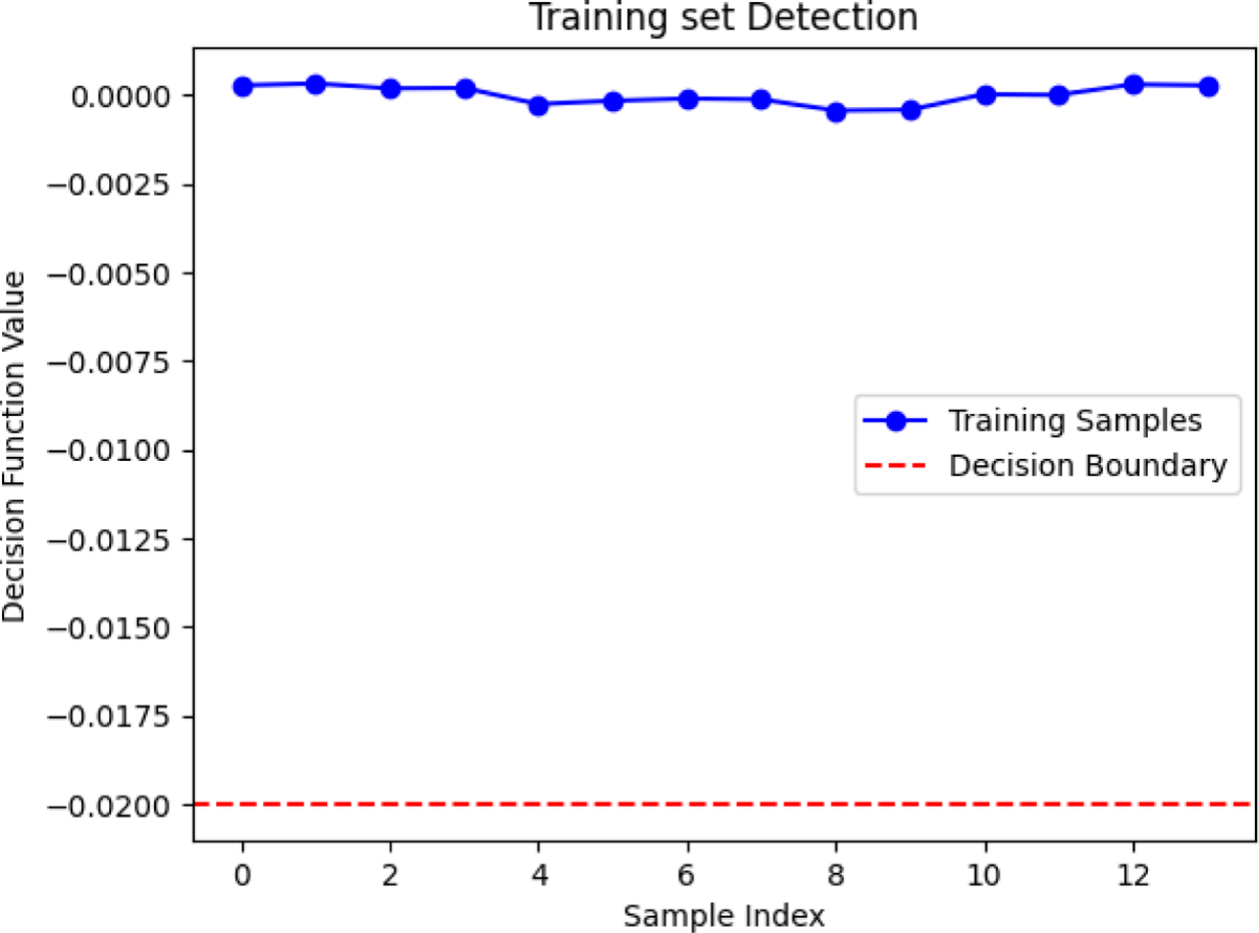
Decision function values of the training set of DMMP (MLDF).

Regarding the MLDF, based on the model’s accuracy, the linear kernel has been proven to be the most effective approach to recognize the inliers. For the DMMP, a threshold of −0.02 was set up (Figure 4), meanwhile, for acetone, the threshold of the decision function values was set to −0.10. By employing these thresholds, no false negatives were obtained for both analytes, reaching a model’s accuracy of 100%. This result was evaluated using the *k*-fold cross-validation method. By using a feature extraction process, the variance caused by the concentration of the samples was reduced, leading to more comparable function values among the samples with different concentrations.

Additionally, the time taken to train the models and make predictions using the evaluation set was measured to assess the efficiency of each DF approach. Training the model with the LLDF approach took 0.015 s, while making predictions took 0.0057 s. In contrast, the MLDF approach was faster, requiring only 0.0035 s for training and 0.009 s for prediction.

For the HLDF approach for the TATP, the OC-SVM has been chosen for the IMS, and meanwhile, the SIMCA model has been chosen for the GC-QEPAS sensor. The OC-SVM has been computed using the four different kernel functions as before mentioned. The linear kernel has been proven to be more effective in modeling the IMS plasmagrams, leading toward an accuracy of 100% by setting a decision function threshold of 0.0. On the other hand, the SIMCA method relies on the Euclidean and the Mahalanobis distance to make the classification of new samples. In order to avoid false negatives in safety applications, the distances were set to 5.5 and 6.5, respectively. By employing these parameters, the overall accuracy of the model, evaluated by cross-validation, was 100%. The performance parameters for TATP are shown in Table 6.

Additionally, the time taken to train the models of HLDF and make predictions using the evaluation set was measured to assess the efficiency of OC-SVM on IMS data and SIMCA on GC-QEPAS data. Training the OC-SVM took 0.0022 s, while making predictions took 0.0019 s. In contrast, the SIMCA model was slower, requiring 0.0055 s for training and 0.0032 s for prediction. This demonstrates the effectiveness of the MLDF approach in forensic scenarios, where rapid predictions are crucial. The speed of predictions across all DF approaches highlights their effectiveness in situations where quick results are essential.

## 6 Conclusions and Future Directions

This study has enabled the development of three data fusion (DF) approaches—LLDF, MLDF, and HLDF—using IMS and GC-QEPAS sensors, focusing on three compounds of forensic interest: acetone, DMMP, and TATP. These approaches were designed with the aim of enhancing operator safety during a crime scene investigation. A critical aspect of this research was ensuring accurate placement of sensors within the crime scene. This was achieved through traditional measuring tools and advanced laser distance meters, guaranteeing that the sensors analyzed the same substance on a surface or as a cloud in the air. For indoor scenarios, a distance of less than 1 m along all three axes (*x*, *y*, and *z*) was deemed appropriate for reliable data fusion. Beyond this threshold, the sensors operated independently by using their specific detection methods.

The first DF approach employed LLDF, achieving a high accuracy of 100% by appropriately setting a cutoff for the decision function values. However, the model’s performance was influenced by the concentration of the compound in the sample, particularly at lower concentrations. In contrast, the second approach, MLDF, used PCA for feature extraction to reduce variance associated with sample concentration, resulting in compact sample groupings and improved robustness and reliability. This method also achieved a 100% accuracy. The third approach integrated OC-SVM for IMS data and the SIMCA model for GC-QEPAS data, demonstrating the effectiveness of advanced DF techniques for forensic applications.

A notable feature of these approaches is their rapid prediction capabilities, which are essential for real-time forensic applications. For instance, the MLDF approach demonstrated the highest time efficiency, requiring only 0.0035 s for training and 0.009 s for prediction. These methods underscore the practicality of DF systems in scenarios demanding swift decision-making, such as terrorist attacks involving chemical warfare agents or investigations of illegal manufacturing sites. The automated Python script developed in this study played a pivotal role in ensuring proper sensor synchronization and positioning, enabling accurate DF analysis, and reducing human error.

While the DF approaches demonstrated high accuracy, their reliability depends on target compound concentrations being significantly above the limit of detection (LoD). Accuracy decreases as concentrations approach the LoD, and the lowest concentration of analytes used in this study was over one unit above the LoD. Additionally, the methods were not tested with strong interference concentrations, highlighting areas for future research. The automation facilitated by the Python script ensures precise and efficient data analysis, further enhancing the safety of forensic specialists in potentially hazardous environments.

Despite the promising results, this study has limitations. It focused on three chemical compounds, indoor scenarios, and a limited data set. Outdoor environments pose additional challenges such as wind and weather conditions affecting the analyte cloud. Future research should expand the range of target analytes, increase the number of sensors, and leverage advancements in sensor technology to improve model performance and broaden the applicability of these DF approaches. Moreover, extending the data set used to train these models will enhance their robustness and reliability.

In summary, this research represents a significant advancement in integrating machine learning methodologies into forensic investigations, demonstrating the potential of DF approaches to improve on-site analytical accuracy and operator safety. With ongoing technological and methodological developments, the methods presented in this study hold promise for enhancing forensic investigations across diverse operational contexts.

## Data Availability

The Python-based implementation of the proposed DF system is freely available at the following link: https://github.com/article-git/AI-DataFusion, along with all the data used to conduct this study.

## Abbreviations

BMK: benzyl methyl ketone
CPU: central processing unit
CSI: crime scene investigation
CWA: chemical warfare agents
DEMP: diethyl methyl phosphonate
DES: diethyl sulfide
DF: data fusion
DMMP: dimethyl methylphosphonate
DMMP: dimethyl-methyl phosphonate
DPGME: dipropylene-glycol-methyl-ether
ENFSI: European Network of Forensic Science Institute
FN: false negatives
FP: false positives
GC-QEPAS: gas chromatography-quartz enhanced photoacoustic spectroscopy
HLDF: high-level data fusion
IMS: ion mobility spectrometry
IR: infrared
LDA: linear discriminant analysis
LD: laser diode
LLDF: low-level data fusion
LoD: limit of detection
MEK: methyl ethyl ketone
MEMS: micro-electro-mechanical system
ML: machine learning
MLDF: mid-level data fusion
OC-SVM: one-class support vector machines
PCA: principal component analysis
PDF: probability density function
RM: reduced mobility
RT: retention time
SIMCA: soft independent modeling of class analogy
std.dev.: standard deviation
TATP: triacetone triperoxide
TMP: trimethyl phosphonate
TN: true negatives
TP: true positives
